# Patient-Reported Outcomes of Rotigotine in Parkinson's Disease: Real-World Evidence on Symptom Control and Quality of Life From China

**DOI:** 10.1155/padi/8839543

**Published:** 2025-11-25

**Authors:** Xiaodong Zhu, Hongcan Zhu, Lei Chen, Tanja Heidbrede, Saori Shimizu, Yingyan Zhou, Weiwei Sun, Wenhuan Cheng, Guiyun Cui, Jian Wang

**Affiliations:** ^1^Department of Neurology, Tianjin Medical University General Hospital, Tianjin, China; ^2^Department of Neurology, The First Affiliated Hospital of Zhengzhou University, Zhengzhou, Henan, China; ^3^Department of Neurology, Tianjin Huanhu Hospital, Tianjin, China; ^4^Global Real World Evidence, UCB BioSciences, Monheim Am Rhein, Germany; ^5^Global Clinical Development, UCB Pharma, Brussels, Belgium; ^6^Global Developed Brands, UCB Pharma, Brussels, Belgium; ^7^Biometrics & Data Science, UCB Pharma, Shanghai, China; ^8^Global Pharmacovigilance, UCB Pharma, Shanghai, China; ^9^Department of Neurology, The Affiliated Hospital of Xuzhou Medical University, Xuzhou, Jiangsu, China; ^10^Department of Neurology and National Research Center for Aging and Medicine & National Center for Neurological Disorders, State Key Laboratory of Medical Neurobiology, Huashan Hospital Affiliated to Fudan University, Shanghai, China

**Keywords:** Chinese, dopamine agonist, drug safety, Parkinson's disease, quality of life, rotigotine, transdermal patch

## Abstract

**Background:**

Parkinson's disease (PD) is a progressive neurodegenerative disorder impacting the quality of life, with a notable prevalence worldwide, including China. Rotigotine, a silicone-based patch that transdermally delivers a dopamine agonist, has shown promise as a PD treatment option.

**Objective:**

This study aimed to evaluate the safety and effectiveness of rotigotine in a real-world Chinese population.

**Method:**

This multicenter, prospective, noninterventional postmarketing surveillance study was conducted across 27 hospitals in China from March 2021 to June 2023. It included adult patients with early and advanced idiopathic PD, newly initiating rotigotine (includes receiving rotigotine up to four weeks prior to enrollment). Safety was assessed through treatment-emergent adverse drug reactions (ADRs), serious adverse events (AEs) (SAEs), and treatment discontinuations due to AEs. Effectiveness was evaluated using the Patient Global Impression of Change (PGIC), Wearing-Off Questionnaire-9 (WOQ-9), and PD Questionnaire-8 (PDQ-8).

**Results:**

Of 829 enrolled patients, 803 were included in the safety set and 572 in the full analysis set. The study reported a safety profile consistent with previous studies, with the most common AE being application site pruritus. The incidence of ADRs was 17.6%, lower than in previous Chinese Phase 3 studies and a Japanese noninterventional study. Over half of the patients reported improvement in PD symptoms as per PGIC, and PDQ-8 scores indicated an overall improvement in quality of life, particularly in patients with advanced PD.

**Conclusions:**

This study reaffirms the safety and effectiveness of rotigotine in a real-world Chinese PD population, including both early and advanced stages, aligning with previous research findings.

## 1. Introduction

Parkinson's disease (PD) is a complex neurodegenerative disorder known for significantly impacting patients' quality of life [1, 2]. Characterized by a range of motor and nonmotor symptoms, PD is rooted in the progressive loss of dopaminergic neurons [3, 4].

In China, PD's prevalence is notable, with an estimated incidence rate of 10.4/100,000 annually and a prevalence of 3.9% among individuals over 50 years old [5]. Globally, the number of people with PD doubled to over 6 million from 1990 to 2015 and is expected to double again to over 12 million by 2040 [6]. It is predicted that by 2030, China could have nearly half of the world's PD population [5].

Rotigotine was introduced as a beacon of hope in this therapeutic landscape [7, 8]. As a dopamine agonist delivered via a silicone-based transdermal patch, rotigotine provides continuous medication over 24 h [9, 10]. Its noninvasive administration method has been globally acknowledged [11, 12], with regulatory approval for its use in both early and advanced PD, as monotherapy or in combination with levodopa [13–16]. Over the past two decades, 7896 study participants have been exposed to rotigotine as part of the clinical development program without any significant risks [17–21].

In real-world settings across multiple countries, the safety and effectiveness of rotigotine for PD patients have been solidly demonstrated [22–24]. This study is the first to assess the safety, effectiveness, and use of the rotigotine transdermal patch in a real-world setting in China for both early and advanced PD.

## 2. Materials and Methods

### 2.1. Study Design

This study is a multicenter, prospective, noninterventional, postmarketing surveillance conducted in China, which aimed to assess the safety and effectiveness of the rotigotine patch in adult patients with early or advanced idiopathic PD in real-world clinical practice. The study was approved by the Institutional Review Board of Huashan Hospital in Fudan University (KY-2020-1118). Patient's data consent was obtained and documented in accordance with local regulations and the ethical principles that have their origin in the principles of the Declaration of Helsinki.

### 2.2. Recruitment and Participants

The recruitment period spanned from Mar 2021 to Mar 2023, and the follow-up period spanned from Mar 2021 to Jun 2023. Totally, 829 patients with early or advanced PD were recruited from 27 hospitals across China. Adult patients (≥ 18 years) diagnosed with idiopathic PD who had been receiving rotigotine patch treatment for a maximum of 4 weeks or who started rotigotine patch at enrollment were recruited. Patients were to be excluded from the study if any of the following criteria applied: (1) Any reason that, in the treating physician's opinion, made the patient unsuitable to participate in this study and (2) the patient had been receiving the rotigotine patch for more than 4 weeks prior to study enrollment.

### 2.3. Treatment Schedule

In this study, PD was managed as per routine clinical practice, which remained unaffected by the study's procedures. The decision to prescribe the rotigotine patch was made in accordance with local medical guidelines and the specific labeling instructions of the product. Patients completed visits approximately every 4 weeks, over a course of 12 weeks from the first dose of the study drug (up to 4 visits). Visit 1 (Week 0) served as the enrollment visit, and then the patients were monitored for up to 12 weeks from the initiation of rotigotine patch treatment or until early termination. Early termination visits (ETVs) were performed for patients discontinuing the rotigotine patch.

### 2.4. Assessment of Safety Variables

Primary variables of this study included treatment-emergent (TE) adverse drug reactions (ADRs) and treatment-emergent serious adverse events (AEs) (SAEs), while secondary safety variables focused on TEAEs and ADRs that led to the discontinuation of the rotigotine patch. An AE/ADR was considered TE if it had an onset or had worsened on or after the date of the first dose of rotigotine patch, but not beyond 30 days following the date of the last rotigotine patch removal within the observational period of this study. Data were collected, and AEs were assessed according to the seriousness and causality of the event, as noted by the physician, and any causal relationship to rotigotine that could not be ruled out.

### 2.5. Assessment of Secondary Variables for Effectiveness

Effectiveness information was collected via self-administered questionnaires, which were validated in Chinese and collected on paper forms. Effectiveness variables of this study were Patient Global Impression of Change (PGIC) rating from the first dose of rotigotine patch to Week 12 or ETV, Wearing-Off Questionnaire-9 (WOQ-9) response (positive/negative) at all visits, and PD Questionnaire-8 (PDQ-8) score at all visits.

In this study, the PGIC scale [25] was utilized to measure patients' self-perceived functional changes since initiating rotigotine patch treatment, with ratings from 1 (*very much improved*) to 7 (*very much worse*). The WOQ-9 [26, 27] was employed to assess changes in both motor and nonmotor symptoms associated with medication “wearing off.” This tool specifically focused on symptom presence and improvement following each dose of a PD treatment. In addition, the PDQ-8 [28, 29] was used to gauge health-related quality of life across eight key domains, including mobility, daily activities, emotional well-being, and others. The PDQ-8's scoring system, ranging from 0 to 100, provided a comprehensive overview of the patients' overall health status, where lower scores indicated better quality of life.

### 2.6. Assessment of Other Variables

Patient demographics and clinical characteristics (age [years], gender [M/F], geographic region [city], education [highest level], ethnic origin, height [m], and body weight [kg]), disease characteristics (age at onset [years] of PD, initial symptom [description], family history, and Hoehn and Yahr staging [original version [30], performed during the on-stage]), and treatment regimen (dose, frequency, type of dosing, start and withdrawal time of rotigotine patch, and all other concomitant medications) information were also collected.

### 2.7. Statistical Analyses

All analyses in this study were descriptive. AEs were coded by primary System Organ Class (SOC) and preferred term (PT) using the most recently available Medical Dictionary for Regulatory Activities (MedDRA) (Version 26.0). The safety set (SS) comprises all participants who received at least one application of the rotigotine patch. This set was used for safety analyses and study drug exposure summaries. The full analysis set (FAS) comprised participants who started rotigotine patch treatment at enrollment and completed at least one postdose effectiveness assessment. Those who had been on the rotigotine patch for up to 4 weeks before enrollment were excluded from the FAS. The FAS was used for demographic analyses and effectiveness analyses.

SAS Version 9.3 was used for analysis. For both categorical and continuous parameters, descriptive statistics were applied. The denominator for percentages was based on the number of patients appropriate for the purpose of analysis.

## 3. Results

### 3.1. Patient Disposition

**Figure 1:**
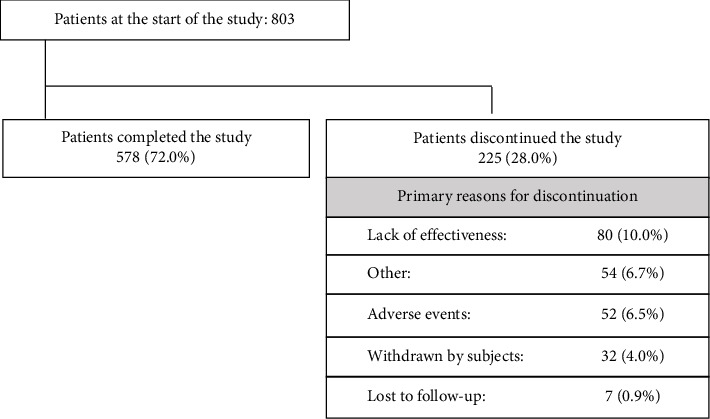
Patient disposition and discontinuation reasons in the safety set. Patient disposition and main reasons for study discontinuation in the safety set. A completed study was defined as completing the last scheduled visit within the study period. Percentages are based on the overall population.

Of the 829 patients screened, 26 failed the screening, and 803 were enrolled. As a result, the SS consisted of 803 patients, and the FAS consisted of 572 patients. Out of the 803 patients in the SS, 578 (72.0%) completed the study, while 225 (28.0%) discontinued the study (Figure 1). The main reasons for study discontinuation, with incidences of 5% or higher, were lack of effectiveness (10.0%), other reasons (6.7%), and AEs (6.5%). The primary reasons for discontinuation of treatment with the rotigotine patch (≥ 5%) were lack of effectiveness (10.3%) and AEs (6.4%).

### 3.2. Demographics and Clinical Characteristics

Table 1 presents the baseline demographic and clinical characteristics of patients within the FAS, categorized by PD stage as assessed by the treating physician. At baseline, the mean age of all patients was 64.4 years. Notably, the advanced PD group exhibited a significantly higher proportion of patients with a familial history of PD (8.9%, nearly twice the 4.9% observed in the early PD group) and experienced the on–off phenomenon (73.2% compared to 39.4% in the early PD group). Resting tremor and bradykinesia emerged as the most prevalent initial symptoms among both early and advanced PD patients.

### 3.3. Exposure to Rotigotine

The mean duration that patients in the SS received rotigotine patch during this study was 71.3 (SD = 26.7) days, and the mean daily dosage was 3.42 mg, with a SD of 1.19 mg. Overall compliance with study medication during the exposure period was approximately 99%.

### 3.4. Safety and Tolerability

#### 3.4.1. TEAEs

Among these, 154 patients (71.3%) had mild events, 49 patients (22.7%) had moderate events, and 13 patients (6.0%) had severe events. The most frequently reported TEAEs were application site pruritus (6.5%), nausea (3.0%), and dizziness (2.7%).

#### 3.4.2. ADRs

Of the 141 (17.6%) patients with ADRs, 112 (79.4%) had mild events, 25 (17.7%) had moderate events, and 4 (2.8%) had severe events. The most common ADR was application site pruritus (6.5%), which was also the most common ADR leading to withdrawal. Gastrointestinal disorders were the 2nd frequently reported ADRs (4.0%). In the SS, the percentages of patients with TE ADRs were uniformly distributed by age (20.5% in 18, < 50 years; 19.8% in 50, < 65 years; 14.9% in 65, < 75 years; and 18.3% in ≥ 75 years) and by gender (18.3% male, 16.7% female) (Table 2).

#### 3.4.3. TE SAEs

In the SS, 25 SAEs were reported by 19 of 803 patients (2.4%), with only 1 SAE (hallucination) related to the study drug. The most frequently reported SAEs by PT were COVID-19 and worsening of PD (0.2% each).

#### 3.4.4. Application Site Reactions as ADRs

Application site reactions were reported in 94 patients (11.7%) with 100 events, all nonserious. The most frequently reported application site reaction (also the most frequently reported ADR) was application site pruritus (6.5%), followed by application site dermatitis (1.9%) and application site erythema (1.5%).

### 3.5. Effectiveness

#### 3.5.1. PGIC–First Dose to Last Visit

**Figure 2:**
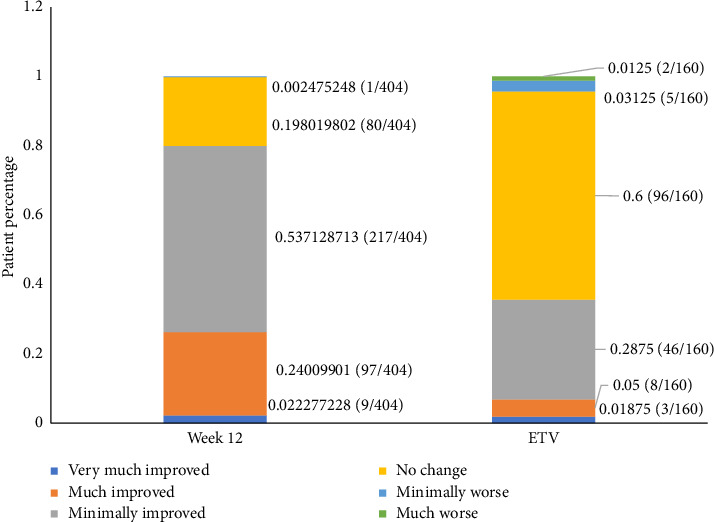
PGIC results by Week 12 of an ETV (full analysis set). PGIC from baseline to Week 12 and from baseline to early termination visit. Data labels with a value of “0%” are not displayed in the figure. PGIC: Patient Global Impression of Change; ETV: early termination visit.

At Week 12, among patients who completed the study and with nonmissing results, 79.9% reported at least minimal improvement in PD symptoms from baseline in the PGIC assessment, 19.8% reported no change, and 0.2% reported minimal worsening; no patients reported much worse or very much worse symptoms (Figure 2). Most patients (60.0%) who discontinued the study reported no change in symptoms at the ETV, > 30% reported varying degrees of improvement, while < 5% reported worsening. These results were similar when comparing patients with early PD to those with advanced PD.

#### 3.5.2. WOQ-9 Responses

From baseline to Week 4, 391 (69.4%) patients stayed positive, 59 (10.5%) patients stayed negative, 31 (5.5%) patients changed from positive to negative, and 82 (14.6%) patients changed from negative to positive. Similar results were observed for patients from baseline to Weeks 8 and 12 (Table 3). The percentages of patients changing from positive at baseline to negative were similar between early (5.2%–6.3%) and advanced (2.7%–4.0%) PD stages. Similar results were observed for patients changing from negative to positive.

#### 3.5.3. PDQ-8

**Figure 3:**
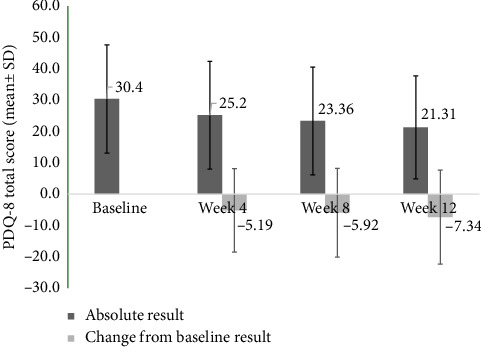
PDQ-8 total score and change from baseline (full analysis set). Total score and change from baseline by visit in the PDQ-8. PDQ-8: Parkinson's disease Questionnaire-8.

Results from the total score of the PDQ-8 showed improvement after initiating rotigotine and continued to improve from baseline to Week 12 (Figure 3). Advanced PD patients showed numerically greater improvement than early-stage patients (−8.16 vs. −6.87 at Week 12). Both motor and nonmotor scores improved, with numerically larger improvements seen in advanced PD patients (−0.6 vs. −0.5 for motor, and −2.0 vs. −1.7 for nonmotor scores at Week 12). For motor score, improvement during Week 8 to Week 12 was stable, with a mean change of about −0.5. While for the nonmotor score, the improvement continued to increase from baseline to Week 12 (changes from baseline result were −1.4 at Week 4, −1.6 at Week 8, and −1.8 at Week 12).

## 4. Discussion

This study represents the first real-world evidence (RWE) for rotigotine patches in China's patient population with both early and advanced PD. Previously, two Phase 3 trials in China [13, 14] assessed rotigotine's efficacy and safety in early and advanced PD. In the broader Asian context, a Japanese noninterventional study (NIS) [22] has provided similar data for rotigotine. The insights gleaned from this study hold significant potential for refining future therapeutic approaches and dosing protocols, particularly in the context of the Chinese patient population.

Our findings corroborate the safety profile of rotigotine in real-world settings, aligning with outcomes from the two prior Chinese Phase 3 studies. Application site pruritus was the most common AE and the leading reason for discontinuation, accounting for 1.7% of TE ADRs that led to treatment cessation. The predominant SAEs were COVID-19 and PD worsening, each at 0.2%. Conducted amidst the COVID-19 pandemic, these results were anticipated, and no novel safety concerns emerged, maintaining consistency with previous rotigotine studies.

Notably, the ADR incidence rate in this study (17.6%) was lower than that in the Chinese Phase 3 studies (36.3% and 38.7%) and the Japanese NIS (34.3%). The serious ADR rate (0.1%) mirrored the Japanese NIS (0.2%), while SAE occurrence (2.4%) was slightly less than that in the Chinese Phase 3 studies (4.8% in early PD and 3.4% in advanced PD). The discrepancies may stem from variances in study design, dosages, patient demographics, and outcome metrics. For example, the average daily dose of rotigotine used in this study (3.42 mg) was reflective of clinical practice in China, but is lower than that used in the Phase 3 trials for advanced PD (11.8 mg) and the Japanese NIS (4.5 mg), and the recommended dose outlined in the package insert of rotigotine. Therefore, comparisons should be approached with caution. It reinforces the message that, particularly in the context of real-world clinical settings, the appropriate dosing is key to optimizing patient outcomes.

Due to a unique drug delivery system, we have observed application site reaction as the most common ADR; however, gastrointestinal disorders were notably low (4.0%) in this study, much lower than with other dopamine agonists [31, 32]. In Chinese clinical practice, the transdermal rotigotine patch is sometimes chosen as a substitute for oral dopamine agonists in PD patients who cannot tolerate the gastrointestinal adverse effects of these medications. This clinical preference mirrors rotigotine's capacity to deliver therapeutic benefits comparable to other agents while reducing gastrointestinal side effects.

Data from the PGIC showed that more than half of the participants (79.9%) reported a perceptible improvement in their condition following the initiation of treatment. Specifically, 53.7% of patients reported minimal improvement, 24.0% much improvement, and 2.2% very much improvement, underscoring the treatment's effectiveness from the patient's perspective, providing a key metric for evaluating the success of the therapy in a real-world setting.

Analysis of PDQ-8 scores across the study cohort indicated a consistent trend of improvement in overall patient condition, extending from the onset of the treatment to the 12-week endpoint. This improvement was more pronounced in patients with advanced PD, highlighting the treatment's potential impact in more severe cases. The data reflect a comprehensive enhancement in both motor and nonmotor symptoms, underscoring the broad therapeutic effectiveness of rotigotine over the course of the study.

In the analysis of WOQ-9 responses, a majority of patients who initially tested positive for wearing-off symptoms continued to do so in follow-up visits. Intriguingly, a considerable number of patients who initially did not exhibit these symptoms reported positive indications in subsequent assessments (Week 4–Week 12). These observations might not only reflect the progressive nature of PD but could also be influenced by subjective factors such as patient self-reporting or adjustments in treatment regimens typical of real-world clinical settings. While the WOQ-9 serves as an effective tool for screening and identifying wearing-off symptoms, it should not be considered a definitive diagnostic instrument [26]. Its reliance on patient self-reporting introduces a degree of subjectivity that could lead to potential biases.

This study, while providing valuable insights, is not without its limitations. Its observational design led to variability in treatment duration and follow-up, potentially introducing recall bias, as patient assessments may have been disproportionately influenced by their most recent experiences. In addition, the discretion exercised by physicians in diagnosing and selecting treatments might have resulted in confounding due to the preferential inclusion of certain patient subgroups. Furthermore, the overlap of the COVID-19 pandemic with the study's recruitment phase might have impacted the patient demographics and baseline disease characteristics, an effect that was not explicitly analyzed within the study's framework.

## 5. Conclusions

In this multicenter, prospective, observational study, we recruited 829 Chinese patients with idiopathic PD in both early and advanced stages; our study conclusively demonstrates that the rotigotine patch maintains a well-tolerated safety profile while also proving its effectiveness in this patient cohort. These results are consistent with previous studies on rotigotine, with no new safety concerns emerging. This study underscores the potential of rotigotine as an effective treatment option for PD, affirming its safety and effectiveness in a diverse range of real-world settings.

## Figures and Tables

**Table 1:** Demographic and baseline characteristics by PD stage (full analysis set).

Items	All subjects *N* = 572	Stage of PD	
		Early (*n* = 348)	Advanced (*n* = 224)
Age (years)			
Mean (SD)	64.4 (9.3)	63.7 (9.5)	65.5 (8.8)
Min, max	27, 94	27, 87	39, 94
Female, *n* (%)	263 (46.0)	149 (42.8)	114 (50.9)
BMI (kg/m^2^)	569	347	222
Mean (SD)	23.51 (3.12)	23.69 (2.95)	23.23 (3.36)
Min, max	14.9, 33.1	14.9, 33.1	15.3, 32.6
Ethnic subgroup, *n* (%)			
Han	564 (98.6)	34.2 (98.3)	222 (99.1)
Not Han	8 (1.4)	6 (1.7)	2 (0.9)
Family history of Parkinson's disease, *n* (%)	37 (6.5%)	17 (4.9)	20 (8.9)
On–off phenomenon, *n* (%)			
Yes	301 (52.6)	137 (39.4)	164 (73.2)
No	271 (47.4)	211 (60.6)	60 (26.8)
Hoehn and Yahr stage, *n* (%)			
0	6 (1.0)	5 (1.4)	1 (0.4)
1	100 (17.5)	96 (27.6)	4 (1.8)
2	255 (44.6)	211 (60.6)	44 (19.6)
3	157 (27.4)	32 (9.2)	125 (55.8)
4	48 (8.4)	4 (1.1)	44 (19.6)
5	6 (1.0)	0	6 (2.7)
Initial Parkinson's disease symptoms, *n* (%)			
Resting tremor	372 (65.0)	216 (62.1)	156 (69.6)
Bradykinesia	343 (60.0)	214 (61.5)	129 (57.6)
Rigidity	164 (28.7)	92 (26.4)	72 (32.1)
Postural instability and gait difficulty	158 (27.6)	83 (23.9)	75 (33.5)
Nonmotor symptoms	97 (17.0)	59 (17.0)	38 (17.0)
Parkinson's disease duration, mean (SD) (years)	5.60 (4.77)	4.28 (3.79)	7.64 (5.38)
Age at onset of Parkinson's disease, mean (SD) (years)	58.3 (9.6)	59.0 (9.9)	57.1 (8.9)

*Note: n*, number of patients; *N*, all patients; Max, maximum; Min, minimum.

Abbreviations: BMI, body mass index; SD, standard deviation.

**Table 2:** Incidence of treatment-emergent ADRs occurring in ≥ 1% of patients by preferred term (safety set).

MedDRA 26.0 System Organ Class Preferred term	All subjects *N* = 803 *n* (%) [#]
Any treatment-emergent ADR	141 (17.6) [211]
Gastrointestinal disorders	32 (4.0) [38]
Nausea	18 (2.2) [20]
Vomiting	10 (1.2) [10]
General disorders and administration site conditions	87 (10.8) [110]
Application site pruritus	52 (6.5) [55]
Application site dermatitis	15 (1.9) [16]
Application site erythema	12 (1.5) [13]
Nervous system disorders	23 (2.9) [26]
Dizziness	11 (1.4) [12]
Skin and subcutaneous tissue disorders	14 (1.7) [17]
Pruritus	9 (1.1) [9]

*Note:* Percentage was calculated as the number of patients in each category divided by all patients (*N* = 803). MedDRA, Medical Dictionary for Regulatory Activities; *n*, number of patients reporting at least 1 AE in that category; [#], the number of individual occurrences of the AE in that category.

Abbreviation: ADR, adverse drug reaction.

**Table 3:** WOQ-9 available results shift from baseline (full analysis set).

	WO results	Baseline		
		Positive *n* (%)	Negative *n* (%)	Total *n* (%)
Week 4	Positive	391 (69.4)	82 (14.6)	473 (84.0)
	Negative	31 (5.5)	59 (10.5)	90 (16.0)
	Total	422 (75.0)	141 (25.0)	563 (100)

Week 8	Positive	330 (69.3)	85 (17.9)	415 (87.2)
	Negative	28 (5.9)	33 (6.9)	61 (12.8)
	Total	358 (75.2)	118 (24.8)	476 (100)

Week 12	Positive	285 (69.2)	72 (17.5)	357 (86.7)
	Negative	24 (5.8)	31 (7.5)	55 (13.3)
	Total	309 (75.0)	103 (25.0)	412 (100)

*Note:* Percentages are based on patients with nonmissing and available WOQ-9 data. Baseline is defined as the last available result prior to the first dose of the study drug.

Abbreviations: WO, wearing off; WOQ-9, Wearing Off Questionnaire-9.

## Data Availability

The data supporting the findings of this study are available on request from the corresponding author. The data are not publicly available due to privacy or ethical restrictions.
